# Acalabrutinib: A Selective Bruton Tyrosine Kinase Inhibitor for the Treatment of B-Cell Malignancies

**DOI:** 10.3389/fonc.2021.668162

**Published:** 2021-05-14

**Authors:** Hussein A. Abbas, William G. Wierda

**Affiliations:** ^1^ Division of Cancer Medicine, Medical Oncology Fellowship, The University of Texas MD Anderson Cancer Center, Houston, TX, United States; ^2^ Department of Leukemia, The University of Texas MD Anderson Cancer Center, Houston, TX, United States

**Keywords:** acalabrutinib, Bruton tyrosine kinase inhibitor, B-cell malignancies, chronic lymphocytic leukemia, mantle cell lymphoma, Waldenstrom’s macroglobulinemia

## Abstract

Bruton tyrosine kinase (BTK) is a validated target for treatment of B-cell malignancies, and oral inhibitors of BTK have emerged as a standard of care for these diseases. Acalabrutinib is a second generation, highly selective, potent, covalent BTK inhibitor that exhibits minimal off-target activity in *in vitro* assays, providing the potential to improve tolerability over the first-in-class BTK inhibitor, ibrutinib. Acalabrutinib was approved for the treatment of relapsed/refractory mantle cell lymphoma (MCL) and chronic lymphocytic leukemia (CLL) in the US in 2017 and 2019, respectively. Acalabrutinib is also undergoing trials for other B-cell malignancies, both as monotherapy and in combinations. In this review, we discuss results from clinical trials evaluating the efficacy and safety of acalabrutinib in patients with CLL, MCL, and Waldenstrom’s macroglobulinemia. Recent phase 3 data showed that acalabrutinib improved progression-free survival (PFS) compared with rituximab plus idelalisib or rituximab plus bendamustine in patients with relapsed/refractory CLL, and acalabrutinib with or without obinutuzumab improved PFS compared with chlorambucil plus obinutuzumab in patients with treatment-naïve CLL. Overall, acalabrutinib had a tolerable safety profile, with most adverse events being grade 1/2 severity (most commonly headache and diarrhea) and a low rate of discontinuation due to adverse events.

## Introduction to Bruton Tyrosine Kinase Inhibitors

Non-Hodgkin lymphoma (NHL) is the most common hematological malignancy, accounting for approximately 4% of all cancers in the US (1). B-cell malignancies, such as diffuse large B-cell lymphoma (DLBCL) and chronic lymphocytic leukemia (CLL)/small lymphocytic lymphoma (SLL), account for most NHL cases (2). Bruton tyrosine kinase (BTK), an essential part of the B-cell receptor signaling pathway, is required for the survival and proliferation of normal (3, 4) and malignant (5) B cells. Accordingly, BTK emerged as an important therapeutic target in B-cell malignancies (6, 7).

The first developed BTK inhibitor, ibrutinib, was approved by the US Food and Drug Administration (FDA) in 2013 for patients with relapsed/refractory mantle cell lymphoma (MCL) who had received ≥1 prior therapy. Ibrutinib was subsequently approved for patients with CLL/SLL (as monotherapy or combined with obinutuzumab), Waldenstrom’s macroglobulinemia (WM), marginal zone lymphoma (MZL; who require systemic therapy and had received ≥1 prior anti-CD20 therapy), and chronic graft *versus* host disease (cGVHD; after failure of ≥1 systemic therapies for GVHD) (8). Ibrutinib monotherapy achieved very high response rates but few, if any, complete remissions and required continuous treatment until disease progression or unacceptable toxicity.

Ibrutinib treatment yielded high response rates (68% in relapsed/refractory MCL; 93% in untreated CLL) and was a major therapeutic advance, with less associated toxicity than seen with chemotherapy (9, 10). Phase 3 trial data showed superior progression-free survival (PFS) *versus* chemoimmunotherapy in patients with CLL who were ≥65 years (87 *versus* 74% with PFS after 2 years) and ≤70 years (89 *versus* 73% PFS after 3 years) (10, 11). Improved overall survival (OS) was reported in two trials evaluating ibrutinib *versus* chlorambucil monotherapy (RESONATE-2) and *versus* a chemoimmunotherapeutic regimen of fludarabine, cyclophosphamide, and rituximab (ECOG E1912) (11, 12).

Ibrutinib-associated adverse events (AEs) included atrial fibrillation and hemorrhage (13, 14). In recent meta-analyses, investigators reported risk ratios of 4.69 [95% confidence interval (CI): 2.17–7.64] for atrial fibrillation and 2.82 (95% CI: 1.52–5.23) for hypertension with ibrutinib treatment (15), plus a higher relative risk of overall bleeding in patients receiving ibrutinib (2.72, 95% CI: 1.62–4.58) *versus* alternative therapy (13). AEs associated with ibrutinib have led to treatment interruption and long-term discontinuation. Some treatment-associated toxicities may be explained by inhibition of kinases other than BTK, including Tec, epidermal growth factor (EGF) receptor, and interleukin-2-inducible T-cell kinase (16). In clinical practice, 24% of patients discontinued ibrutinib by 4 years on treatment, owing to intolerance (17), leaving an unmet need for BTK inhibitors with improved safety and tolerability. Second-generation BTK inhibitors with improved selectivity may address some of the associated toxicity issues. Additionally, mechanisms of resistance *via* mutations downstream of BTK indicate a need for new treatments (18).

Acalabrutinib received FDA accelerated approval in 2017 for the treatment of patients with MCL who had received ≥1 prior therapy (19) and was recently approved for adults with previously untreated or relapsed/refractory CLL based on ELEVATE-TN and ASCEND trials (20–22). Outcomes and safety for acalabrutinib and ibrutinib in patients with MCL, CLL, and WM are listed in **Table 1**; results from direct comparisons of these two agents are currently unavailable. Other covalent BTK inhibitors are in development. Zanubrutinib (BGB-3111) has greater BTK selectivity than ibrutinib with minimal off-target *in vitro* inhibition (31). Zanubrutinib was efficacious in clinical trials of patients with MCL and CLL/SLL, was recently FDA approved for MCL treatment (32), and is being investigated in patients with WM (33–36). Another BTK inhibitor, spebrutinib (CC-292), impaired CLL cell proliferation and improved control of CLL progression when given concurrent with bendamustine in preclinical models (37). However, in a phase 1 study, spebrutinib monotherapy had a shorter duration of response than those reported for ibrutinib or acalabrutinib, although it was well tolerated (38–40). Tirabrutinib (ONO-4059/GS-4059) was investigated in a phase 2 trial for patients with relapsed/refractory CLL combined with idelalisib, entospletinib, and obinutuzumab (41). Basic properties of approved covalent BTK inhibitors are listed in **Table 2**. Third-generation BTK inhibitors, involving noncovalent and reversible inhibitory mechanisms, are in development and include pirtobrutinib (LOXO-305) (44). In this article, we focus on clinical data obtained for acalabrutinib.

**Table 1:** Associated toxicities and treatment outcomes reported with BTK inhibitors.

	Acalabrutinib				Ibrutinib			
	R/R MCL (23, 24)	R/R CLL (22)	TN & R/R WM^a^ (25, 26)	TN CLL (21)	R/R MCL (9)	R/R CLL (end of trial/long-term follow-up) (27, 28)	R/R WM (29)^a^	TN CLL (end of trial/long-term follow-up) (12, 30)
*Number of patients*	*124*	*155*	*106*	*179/179^b^*	*111*	*195/195*	*63*	*136/136*
*Median duration of treatment (range), months*	*17.3 (0.1–35.1)*	*15.7 (1.1–22.4)*	*N/A*	*27.7 (IQR 25.0–32.8)/(IQR 24.8–33.0)*	*N/A*	*8.6 (0.2–16.1)/41.0 (0.2–71.1)*	*19.1 (0.5–29.7)*	*17.4 (0.7–24.7)/57.1 (0.7–66.0)*
*Median duration of follow-up (range), months*	*26.3 (0.3–35.1)*	*16.1 (0.03–22.4)*	*27.4 (IQR 26.0–29.7)*	*28.3 (IQR 25.6–33.1)*	*15.3 (1.9–22.3)*	*9.4 (0.1–16.6)/65.3 (0.3–71.6)*	*N/A*	*18.4/60 (0.1–66)*
**Adverse events (all grades unless otherwise stated)**								
Headache	38%	22%	39%	40%/37%	N/A (<15%)	N/A/21%^c^	2%	N/A/N/A
Nausea	19%	7%	23%	20%/22%	31%	G1 24%, G2 6%, G3 2%/36%^c^	N/A	22%/26%
Diarrhea	36%	18%	33%	39%/35%	50%	G1 27%, G2 10%, G3 4%/62%^c^	3%	42%/50%
Fatigue	28%	10%	22%	28%/18%	41%	G1 18%, G2 12%, G3 3%/42%^c^	N/A	30%/36%
Peripheral edema	N/A	N/A	N/A	12%/9%	28%	N/A/24%^b^	N/A	19%/27%
Myalgia	21%	N/A	N/A	N/A	N/A (<15%)	N/A/N/A	N/A	N/A/N/A
Neutropenia	10%	19%	17%	32%/11%	18%	G3 18%/31%^c^	22%	16%/13%^d^
Bleeding events	33%	26%	58%	43%/39%	N/A	N/A/N/A	6%	N/A/N/A
Grade ≥3	2%	1%	3%	2%/2%	5%	N/A/N/A	N/A	N/A/N/A
Cardiac events (all)	10%	N/A	N/A	N/A	N/A	N/A/N/A	N/A	N/A/N/A
Atrial fibrillation	0	5%	5%	3%/4%	N/A	7%/12%	5%	6%/16%
Hypertension	3%	3%	N/A	3%/2%^b^	N/A	N/A/21%	5%	4%^b^/26%
Infections (all)	53%	N/A	N/A	N/A	N/A	N/A/N/A	N/A	N/A/N/A
Grade ≥3	15%	15%		21%/14%	25%	N/A/45%	N/A	N/A/N/A
Discontinuation due to adverse events	8%	11%	7%	11%/9%	7%	7%/16%	10%	9%/28%
**Treatment outcomes**								
Median progression-free survival (95% CI, when available), months	20 (16.5–27.7)	NR	TN NR, R/R NR	NR/NR	13.9 (7.0–NR)	NR/44.1 (38.5–56.2)	N/A	NR/NR
Progression-free survival rate (95% CI, when available)	2-year: 49.0% (39.6–57.8)	12-month: 88% (81–92)	2-year: TN 90% (47–99), R/R 82% (72–89)	2-year: 93% (87–96)/87% (81–92)	N/A	6-month: 88%/60-month: 40%	2-year: 69.1% (53.2–80.5)	18-month: 90%/5-year: 70%
Median overall survival (95% CI, when available), months	NR	NR	TN NR, R/R NR	NR/NR	NR	NR/67.7 (61.0–NE)	N/A	NR/NR
Estimated overall survival rate (95% CI, when available)	2-year: 72.4% (63.5–79.5)	12-month: 94% (89–97)	2-year: TN 92% (54–99), R/R 89% (80–94)	2-year: 95% (91–97)/95% (90–97)	18-month: 58%	12-month: 90%/N/A	2-year: 95.2% (86.0–98.4)	2-year: 98%/5-year: 83%
Overall response rate	81%	81%	TN 93%, R/R 93%	94%/86%	68%	42.6%/91%	90.5%	86%/92%

BTK, Bruton tyrosine kinase; CI, confidence interval; CLL, chronic lymphocytic leukemia; G, grade; MCL, mantle cell lymphoma; N/A, not available; NE, not evaluable; NR, not reached; R/R, relapsed/refractory; TN, treatment-naïve; WM, Waldenstrom’s macroglobulinemia.

Data are percentages of patients who reported the adverse event, unless stated otherwise.

Please note that there are no head-to-head trials comparing acalabrutinib and ibrutinib—these data come from separate trials, in which patient characteristics differed, so they cannot be compared directly. Data are taken from treatment arms in which acalabrutinib or ibrutinib were given as monotherapy.

a Please note the differences in the trial designs and reporting for the two WM studies: The acalabrutinib trial included both TN and R/R patients with a primary endpoint of ORR and reported all AEs of any grade (regardless of causality), whereas the ibrutinib trial was in R/R patients with a primary endpoint of ORR, and only reported adverse events reported of grade 2–4 that were deemed possibly, probably, or definitely associated with study treatment are reported.

b Data are acalabrutinib plus obinutuzumab/acalabrutinib monotherapy.

c Data estimated from Munir et al., 2019 (28) Figure S6.

d Events grade ≥3 only reported.

**Table 2:** General properties of approved BTK inhibitors.

Property	Acalabrutinib (16, 19)	Ibrutinib (8, 16)	Zanubrutinib (42, 43)
Formulation	* Capsules: 100 mg	* Capsules: 70 and 140 mg	* Capsules: 80 mg
		* Tablets: 140, 280, 420, and 560 mg	
Dosing	* 100 mg every 12h	* MCL and MZL: 560 mg once daily	* 160 mg twice daily or 320 mg once daily
		* CLL/SLL, WM: 420 mg once daily	
Pharmacodynamics	* Median steady state BTK occupancy of ≥95% in peripheral blood; maintained over 12h with 100 mg dose	* >90% BTK active site occupancy in PBMCs up to 24h after ≥2.5 mg/kg/day	* Median steady state BTK occupancy of 100% in PBMCs; maintained over 24h with 320 mg daily dose
Pharmacokinetics	* Half-life: 1h	* Half-life: 4–6h	* Half-life: 2–4h
	* Median T_max_: 0.9h	* Median T_max_: 1–2h	* Median T_max_: 2h
	* Main route of elimination: metabolism (mainly by CYP3A); primarily excreted *via* feces	* Main route of elimination: metabolism (mainly by CYP3A); primarily excreted *via* feces	* Main route of elimination: metabolism (mainly by CYP3A); primarily excreted *via* feces
	* No effect of mild or moderate renal impairment on PK (not evaluated in patients with severe renal impairment)	* Renal impairment effects PK	* No effect of mild or moderate renal impairment on PK (not evaluated in patients with severe renal impairment)
Drug interactions	* CYP3A inhibitors: dose modification may be required	* CYP3A inhibitors: dose modification required	* CYP3A inhibitors: modify dose with moderate or strong CYP3A inhibitors
	* CYP3A inducers: avoid co-administration with strong CYPA3 inducers	* CYP3A inducers: avoid co-administration with strong CYPA3 inducers	* CYP3A inducers: avoid co-administration with moderate or strong CYPA3 inducers
	* Gastric acid reducing agents: avoid administration with PPIs; stagger dosing with H2-receptor antagonists and antacids		
Off-target effects (pathway level)	* Minimal	* EGFR family kinases	* Weak ITK
		* Src family kinases	
		* Tec family kinases	
Most common adverse reactions (≥30%) in patients with B-cell malignancies^a^	* Anemia	* Thrombocytopenia	* Neutrophil count decreased
	* Neutropenia	* Diarrhea	* Platelet count decreased
	* Upper respiratory tract infection	* Fatigue	* Upper respiratory tract infection
	* Thrombocytopenia	* Musculoskeletal pain	* White blood cell count decreased
	* Headache	* Neutropenia	
	* Diarrhea	* Rash	
	* Musculoskeletal pain	* Anemia	
		* Bruising	
Clinically significant adverse reactions^a^	* Major hemorrhage^b^ (3%)	* Major hemorrhage^b^ (4%)	* Grade ≥3 bleeding events (2%)
	* Serious or grade ≥3 infections (19%)	* Grade ≥3 infections (21%)	* Grade ≥3 infections (23%)
	* Grade 3 or 4 neutropenia (23%)	* Grade 3 or 4 neutropenia (23%)	* Grade 3 or 4 neutropenia (27%)
	* Grade 3 or 4 anemia (8%)	* Grade 3 or 4 thrombocytopenia (8%)	* Grade 3 or 4 thrombocytopenia (10%)
	* Grade 3 or 4 thrombocytopenia (7%)	* Grade 3 or 4 anemia (3%)	* Grade 3 or 4 anemia (8%)
	* Grade 3 or 4 lymphopenia (7%)	* Cardiac arrhythmias (grade ≥3 ventricular tachyarrhythmias, 0.2%; grade ≥3 atrial fibrillation and atrial flutter, 4%)	* Second primary malignancies (9%; skin cancer [6%])
	* Second primary malignancies (12%; skin cancer [6%])	* Hypertension (all grades, 19%; grade ≥3, 8%)	* Cardiac arrhythmias (all grades, 2%; grade ≥3, 0.6%)
	* Cardiac arrhythmias (all grades, 4.1%; grade 3, 1.1%)	* Second primary malignancies (other malignancies, 10% [including non-skin carcinomas, 4%]; non-melanoma skin cancer, 6%)	

BTK, Bruton tyrosine kinase; CLL, chronic lymphocytic leukemia; CYP3A, cytochrome P450 family 3 subfamily A; EGFR, epidermal growth factor receptor; ITK, interleukin-2-inducible T-cell kinase; MCL, mantle cell lymphoma; MZL, marginal zone lymphoma; PBMC, peripheral blood mononuclear cells; PK, pharmacokinetics; SLL, small lymphocytic leukemia; WM, Waldenstrom’s macroglobulinemia.

a Adverse reaction data are reported for patients receiving monotherapy and are reported as percentages of patients who reported the adverse reaction.

b Serious or grade ≥3 bleeding or any central nervous system bleeding.

## Acalabrutinib Mechanism of Action and Pharmacokinetics

Acalabrutinib is a highly selective, potent, covalent inhibitor of BTK with minimal off-target activity (16), having a narrower spectrum of kinase inhibition on kinome analysis than observed with ibrutinib. It has a 2-pyridylbenzamide moiety and an electrophilic 2-butynamide moiety that are involved in covalent binding to the cysteine (C)481 of BTK (16). Acalabrutinib had higher *in vitro* kinase selectivity than ibrutinib, zanubrutinib, and spebrutinib, and similar selectivity to tirabrutinib (45). In biochemical assays, ibrutinib inhibited Src family kinases, whereas acalabrutinib and tirabrutinib did not inhibit EGF-induced EGF receptor activation or T-cell receptor-mediated T-cell activation in cellular assays at physiologically relevant concentrations (45). The improved selectivity of acalabrutinib compared with ibrutinib is expected to reduce the occurrence of some ibrutinib-associated AEs. As an example, studies showed reduced platelet dysfunction with acalabrutinib compared with ibrutinib, which may be due to inhibition of Src family kinases by ibrutinib but not by acalabrutinib (46, 47); however, whether this results in fewer bleeding AEs in the clinic is unclear.

In healthy volunteers, acalabrutinib showed rapid absorption (time to maximum plasma concentration, 0.5–1.0 h across dose cohorts up to 100 mg) and fast elimination (half-life, 0.88–2.1 h) (16). Twice-daily dosing of acalabrutinib 100 mg resulted in a median BTK occupancy of 97% before and 99% 4 h after dosing at steady state (40). Reduced absorption and plasma levels may be seen with concurrent proton pump inhibitors (which are therefore contraindicated with acalabrutinib); staggered dosing with antacids and H2-receptor antagonists is recommended (19).

## Clinical Results

### MCL

The approval of acalabrutinib for patients with MCL was based on the ACE-LY-004 open-label, phase 2, single-arm trial conducted in 124 patients with relapsed/refractory MCL. Median age of patients was 68 years, 80% were male, and 93% had an Eastern Cooperative Oncology Group (ECOG) score ≤1 (23). After a median follow-up of 15.2 months, acalabrutinib monotherapy showed a durable high response rate and a favorable safety profile (23). With longer-term follow-up (median, 26 months; range, 0.3–35.1 months), the investigator-assessed overall response rate (ORR) was 81%; 43% of patients achieved complete remission (CR). The median duration of response (DOR) was 26 months, and the median PFS was 20 months (24). Discontinuations were mainly due to disease progression (44%). Minimal residual disease (MRD) was evaluated in 29 of 124 patients, eight of whom achieved CR with undetectable MRD (sensitivity 5 × 10^−6^) on acalabrutinib monotherapy (24).

The most common AEs (any grade) were headache (38%), diarrhea (36%), fatigue (28%), cough (22%), and myalgia (21%). Headaches typically occurred during the first month of treatment, diarrhea generally was limited to the first 6 months, and these AEs usually were grade 1/2. Cardiac AEs occurred in 10% of patients. Four patients (3%) had grade 3/4 events; one of these (acute coronary syndrome) was considered treatment related. There were no cases of new-onset atrial fibrillation. Hypertension occurred in four patients. Bleeding events occurred in 33% of patients [grade 3, 2% (*n* = 3)]. Grade 3/4 infections occurred in 15% of patients; pneumonia was most common (6%) (24). No new safety signals were observed during long-term follow-up.

### CLL

The open-label, multicenter, phase 1/2 ACE-CL-001 trial evaluated acalabrutinib in 61 patients [median age, 62 years; median three prior therapies; 31% del(17p); 75% unmutated immunoglobulin heavy-chain variable gene (IGHV)] with relapsed/refractory CLL (40). After a median of 14.3 months of follow-up (range, 0.5–20 months), 87% of patients remained on acalabrutinib. Discontinuations occurred owing to the investigators’ or patients’ decision (*n* = 8), active autoimmune hemolytic anemia (*n* = 2), fatal pneumonia (*n* = 1), AEs (*n* = 3: diarrhea, gastritis, dyspnea), or disease progression (*n* = 1) (40). The ORR was 95% [partial response (PR), 85%; PR with lymphocytosis, 10%]; no patients achieved CR. In an expansion of this study that included 134 patients, responses were similar after a median follow-up of 41 months (range, 0.2–58 months); 56% of patients remained on treatment, with 21% and 11% of patients discontinuing because of progressive disease or AEs, respectively. CR was achieved in 4% of patients, and Richter transformation occurred in three patients (48).

ACE-CL-001 included a treatment-naïve cohort (*n* = 99; median age, 64 years; 10% del(17p); 62% unmutated IGHV) (49). After 42 months of follow-up (range, 1–48 months), 89% of patients remained on acalabrutinib. Discontinuations were due to AEs (5%: second malignancies, sepsis, urinary tract infection), disease progression (2%), withdrawal of consent (2%), pregnancy (1%), and initiation of subsequent cancer treatment (1%) (49). The ORR was 97%; 5% achieved CR and 92% achieved PR. Median DOR was not reached; the 36-month DOR rate was 98%. Median PFS was not reached; the 36-month PFS rate was 97%. Responses were consistent with longer follow-up (median, 53 months; range, 1–59 months) (50).

Across both cohorts (relapsed/refractory and treatment-naïve), most AEs were grade 1/2 and resolved (48, 49). Diarrhea (relapsed/refractory, 52%; treatment-naïve, 49%), headache (51%; 44%), and upper respiratory tract infection (37%; 40%) were most common. Atrial fibrillation (none led to discontinuation) occurred in 7 and 6% and severe bleeding in 5 and 2% of relapsed/refractory and treatment-naïve patients, respectively (48, 49).

Results from two global, randomized, multicenter, open-label phase 3 trials were recently published. ASCEND (ACE-CL-309) was conducted in 310 patients with relapsed/refractory CLL comparing acalabrutinib *versus* investigator’s choice (idelalisib plus rituximab or bendamustine plus rituximab) (22). At the median follow-up of 16.1 months (range, 0.03–22.4 months), median PFS was not reached on acalabrutinib but was significantly longer than that of the comparator group [16.5 months (range, 14.0–17.1 months); hazard ratio (HR), 0.31; 95% CI: 0.20–0.49; *p* < 0.0001]. Most patients in the comparator arm received targeted therapy, and PFS outcomes appeared similar between comparator options. Twelve-month PFS rates were 88% for acalabrutinib and 68% for comparator; respective ORRs were 81 and 75%. PFS was superior for acalabrutinib *versus* comparator across high-risk subgroups, including del(17p) + mutated *TP53* and advanced Rai stage. Outcomes for patients treated with idelalisib plus rituximab were markedly worse in this study compared with previous studies; this was attributed to a lower treatment exposure that was mainly the result of a high rate of discontinuation because of AEs. Mean duration of exposure was 15.7 months for acalabrutinib monotherapy and 11.5 months for idelalisib plus rituximab. AEs most common with acalabrutinib were headache (22%), neutropenia (19%), and diarrhea (18%). Atrial fibrillation occurred in 5% of patients receiving acalabrutinib, and bleeding events occurred in 26% (major hemorrhage, 1%) (22). There were much lower rates of grade 3/4 AEs and AEs leading to discontinuation in the acalabrutinib monotherapy group (45% and 11%, respectively) than in the idelalisib plus rituximab group (86% and 47%, respectively). Safety results for the bendamustine plus rituximab group were similar to those for acalabrutinib monotherapy.

The ELEVATE-TN (ACE-CL-007) study enrolled 535 patients who were randomly assigned (1:1:1) to acalabrutinib plus the anti-CD20 antibody obinutuzumab, acalabrutinib monotherapy, or obinutuzumab plus chlorambucil (21). At median follow-up (28.3 months), the median PFS was not reached with acalabrutinib-obinutuzumab, compared with obinutuzumab-chlorambucil (22.6 months; HR, 0.10; 95% CI: 0.06–0.17; *p *< 0.0001). Acalabrutinib monotherapy was also associated with a superior PFS (median not reached) *versus* obinutuzumab–chlorambucil (HR, 0.20; 95% CI: 0.13–0.30; *p *< 0.0001). The estimated 24-month PFS rates with acalabrutinib–obinutuzumab, acalabrutinib monotherapy, and obinutuzumab–chlorambucil were 93%, 87%, and 47%, respectively. The superior PFS rates of both acalabrutinib-containing arms *versus* obinutuzumab–chlorambucil were consistent across high-risk subgroups, including del(17p). At this time, median OS was not reached in any arm. The median treatment duration was 27.7 months for acalabrutinib-obinutuzumab and acalabrutinib monotherapy and 5.6 months for obinutuzumab–chlorambucil. AEs were similar in the acalabrutinib-containing arms; AEs of interest (acalabrutinib–obinutuzumab or acalabrutinib monotherapy *versus* obinutuzumab–chlorambucil) were atrial fibrillation (any grade: 3% or 4 *versus* 1%), bleeding (any grade/grade ≥3: 43%/2% or 39%/2% *versus* 12%/0%), and hypertension (grade ≥3: 3% or 2 *versus* 3%) (21). Patients in both acalabrutinib-containing arms were treated with a BTK inhibitor until disease progression. Debate continues regarding the contribution of obinutuzumab to the overall outcome in this setting. Currently, the authors would not routinely add CD20 monoclonal antibody (mAb) treatment to a BTK inhibitor-based treatment, either in first-line or the relapsed setting, when a BTK inhibitor is administered until disease progression in patients with CLL.

The phase 1b/2 ACE-CL-003 study also evaluated treatment with acalabrutinib plus obinutuzumab and was conducted in patients with relapsed/refractory (*n* = 26) or treatment-naïve (≥65 years of age; *n* = 19) CLL (51). The ORR was 95% (6% CR) in treatment-naïve patients (median follow-up, 39 months; range, 1–45 months) and 92% (2% CR) in those with relapsed/refractory CLL (median follow-up, 42 months; range, 20–49 months). At 36 months, 94% of treatment-naïve and 88% of relapsed/refractory patients were progression-free. Treatment was discontinued in 11% of treatment-naïve patients [owing to an AE of metastatic squamous cell carcinoma (*n* = 1) and Richter transformation (*n* = 1)] and in 30% of relapsed/refractory patients [owing to AEs (*n* = 4), Richter transformation (*n* = 2), progressive disease (*n* = 1), and death (*n* = 1)]. The most common AEs were upper respiratory tract infection (71%), weight gain (71%), maculopapular rash (67%), cough (64%), diarrhea (62%), headache (56%), nausea (51%), arthralgia (47%), and dizziness (47%). Two relapsed/refractory patients had grade 3 bleeding events (one hematuria and one muscle hemorrhage). One patient experienced grade 3 atrial fibrillation, which did not result in treatment discontinuation (51).

#### Patients With CLL Who Were Intolerant to Ibrutinib

An additional cohort was added to ACE-CL-001 to evaluate patients who were intolerant to ibrutinib due to severe AEs (52). The most common ibrutinib-related AEs reported at trial entry were rash (24%), arthralgia (18%), diarrhea (15%), fatigue (12%), and hemorrhage (12%). Most of the 33 patients had high-risk disease, including Rai stage III or IV (27%), bulky lymph nodes (31%), del(17p) (38%), del(11q) (22%), and unmutated IGHV (81%); additionally, patients were heavily pretreated (median of four prior therapies). After a median of 19.0 months of treatment (range, 0.7–30.6 months), 23 patients remained on acalabrutinib. Ten patients discontinued treatment owing to progressive disease (*n* = 4), AEs (*n* = 3), and physician decision (*n* = 3). The ORR was 76%. Among 25 responders, median DOR and PFS were not reached; 1-year PFS was 83.4%. Acalabrutinib was well tolerated; 72% of the 61 ibrutinib-related AEs associated with intolerance did not recur with acalabrutinib treatment, and 13% recurred at a lower grade (52). This study indicated tolerability and safety for acalabrutinib in ibrutinib-intolerant patients, allowing these patients to continue to receive a BTK inhibitor. Following this, a phase 2 study (ACE-CL-208) was conducted in 60 patients with relapsed/refractory CLL who were ibrutinib-intolerant due to grade 3/4 AEs (including atrial fibrillation/flutter, diarrhea, arthralgia, and rash) after a median treatment duration of 6 months (range, <1–55 months). At the median follow-up of 23 months, 37 patients remained on acalabrutinib. The ORR was 72% (5% CR). Of the 23 patients who discontinued treatment, seven (12%) discontinued due to AEs (53). In the authors’ experience, a lower proportion of patients with CLL require dose reduction or need to stop treatment when on acalabrutinib, compared with ibrutinib. The ability to maintain on-target treatment may improve overall long-term disease control and outcomes. Objective evidence for this hypothesis will require a randomized comparison assessment, which is currently ongoing in a phase 3 study (NCT02477696).

#### Waldenstrom’s Macroglobulinemia

The efficacy and safety of acalabrutinib were investigated in patients with treatment-naïve (*n* = 14) or relapsed/refractory (*n* = 92) WM in a phase 2 trial (ACE-WM-001) (25). Acalabrutinib was highly effective, with an ORR of 93% (median follow-up, 27.4 months; range, 26.0–29.7 months). The 2-year DOR was 90% (95% CI: 47–99%) for treatment-naïve and 82% (95% CI: 72–89%) for relapsed/refractory patients; the respective 2-year PFS rates were 90% (47–99%) and 82% (72–89%), and the respective 2-year OS rates were 92% (95% CI: 54–99%) and 89% (95% CI: 80–94%). Overall, 50% of treatment-naïve and 25% of relapsed/refractory patients discontinued treatment. The most common AEs were headache (39%) and diarrhea (31%). Five patients had atrial fibrillation (grade 3/4, *n* = 1), and three had grade 3/4 bleeding. One treatment-related death (intracranial hematoma) was reported (25).

### Acalabrutinib Combination Therapy in B-Cell Malignancies

Acalabrutinib combined with the phosphatidyl-3-kinase delta (PI3K*δ*) inhibitor ACP-319 was investigated in 40 patients with relapsed/refractory B-cell malignancies. The combined treatment was tolerated with manageable AEs; however, patients with CLL/SLL were switched to acalabrutinib monotherapy, owing to toxicities and limited benefit with added ACP-319 (54). The most common AEs were increased levels of aspartate aminotransferase (48%) and alanine aminotransferase (52%), diarrhea (52%), fatigue (40%), and rash (40%). Twenty-two patients discontinued treatment (5 of 7 owing to AEs with acalabrutinib/ACP-319). No deaths were due to AEs (54).

A single-arm phase 1/2 trial of acalabrutinib combined with the PD-1 mAb pembrolizumab was conducted in 61 patients with relapsed/refractory DLBCL. The ORR was 26% and was similar for germinal center B-cell-like (GCB) DLBCL and non-GCB DLBCL. The median DOR was 6.9 months, with just six patients remaining on study therapy; however, exceptional responses were observed in two patients (DOR, >24 months) (55).

## Safety of Acalabrutinib

In a pooled safety analysis of seven trials of patients (*n* = 610) receiving acalabrutinib monotherapy (701.5 patient–years of exposure), the most common reported AEs were headache (42.3% any cause, 29.2% treatment-related, 1.3% grade ≥3), diarrhea (38.4%, 16.6%, 2.1%), fatigue (23.4%, 7.4%, 1.5%), nausea (23.1%, 9.3%, 1.5%), and contusion (21.6%, 13.4%, 0%), and 35.7% of patients had serious AEs (treatment-related, 9.5%) (56). Grade 5 AEs were reported in 3.6% of patients; pneumonia was the most frequent. Atrial fibrillation occurred in 2.3% of patients (1.0% grade 3), mostly in those with known risk factors. Infections were reported in 61.0% of patients (16.2% grade ≥3); pneumonia was the most frequent.

Many acalabrutinib-associated side effects were grade 1/2 and were easily managed (57). Ibrutinib treatment was associated with an increase in bleeding events through platelet inhibitory mechanisms, partly due to the off-target Tec kinase inhibition (13, 58). Analysis of platelet aggregation showed that acalabrutinib did not result in the platelet dysfunction observed with ibrutinib (46, 47). However, bleeding events were reported with acalabrutinib (**Table 1**). Although no results from head-to-head trials comparing acalabrutinib with ibrutinib or any other BTK inhibitor are available, a summary of the safety profile of each agent in MCL, CLL, and WM is presented in **Table 1**. Findings of a matching-adjusted indirect comparison suggested significantly lower rates of atrial fibrillation and thrombocytopenia with acalabrutinib *versus* ibrutinib (59).

## Mechanism of Acalabrutinib Therapeutic Resistance

A primary reported mechanism of acalabrutinib resistance in CLL is *BTK* mutation (60). In 103 patients with CLL treated with acalabrutinib and routinely screened for *BTK* mutation, mutations developed in 22 (median time from acalabrutinib initiation to detection, 31.6 months). Sixteen patients had progression of CLL; 11 of these patients (69%) had *BTK* C481 mutations (C481S in ten patients, C481R and C481Y in one). Four patients with the *BTK* C481S mutation had coexisting mutations of either *BTK* T474I (*n* = 1), *BTK* C481R (*n* = 1), or *PLCG2* (*n* = 2); these mutations previously were associated with ibrutinib resistance. These data indicate a similar mechanism of resistance for acalabrutinib and ibrutinib.

## Future Studies

**Table 3** summarizes registered clinical trials of patients with hematological malignancies that include acalabrutinib. The ELEVATE RR/ACE-CL-006 randomized, phase 3, open-label study of acalabrutinib *versus* ibrutinib in 533 previously treated patients with high-risk relapsed/refractory CLL has recently completed (NCT02477696). It has been reported that the trial met the primary endpoint of non-inferiority while demonstrating a significantly reduced rate of atrial fibrillation in patients treated with acalabrutinib *versus* those treated with ibrutinib (65).

**Table 3:** Acalabrutinib clinical trials for hematological malignancies.

Trial name (NCT number) or NCT number	Phase	Enrollment^a^	Patient population	Interventions	Status (as of August 2020)	Publication
**MCL**						
ACE-LY-004 (NCT02213926)	2	124	Adults with R/R MCL	Acalabrutinib	Active, not recruiting	(23, 24)
NCT03623373	1	15	Adults with TN MCL	Acalabrutinib + alternating cycles of BR and CR	Active, not recruiting	N/A
ACE-LY-106 (NCT02717624)	1	70	Adults with TN or R/R MCL	Acalabrutinib + BR	Recruiting	(61)
				Acalabrutinib + Ven + R		
ACE-LY-308 (NCT02972840)	3	546	Patients ≥65 years old with TN MCL	Acalabrutinib + BR	Recruiting	N/A
				Placebo + BR		
NCT03863184	2	24	Adults with TN MCL	Acalabrutinib + lenalidomide + R	Recruiting	N/A
NCT03946878	2	50	Adults with R/R MCL	Acalabrutinib + Ven	Recruiting	N/A
NCT04115631	2	369	Adults with TN MCL	BR and high dose CR vs BR,	Recruiting	N/A
				High dose CR + acalabrutinib		
				BR + acalabrutinib		
NCT04402138	50	2	Adults with MCL (treatment to follow blood or bone marrow transplant)	Acalabrutinib	Recruiting	N/A
NCT04189757	2	30	Adults with ibrutinib-intolerant MCL	Acalabrutinib	Not yet recruiting	N/A
NCT04484012	2	36	Adults with R/R MCL	Acalabrutinib + CD19CAR-CD28-CD3zeta-EGFRt-expressing Tn/mem-enriched T-lymphocytes	Not yet recruiting	N/A
**CLL**						
ACE-CL-002 (NCT02157324)	1	12	Adults with R/R CLL	Acalabrutinib + ACP-319	Active, not recruiting	N/A
ACE-CL-003 (NCT02296918)	1	69	Adults with R/R or TN CLL/SLL/PLL	Acalabrutinib + G	Active, not recruiting	(51, 62)
				Acalabrutinib + Ven + R (R/R only)		
				Acalabrutinib + G + Ven (TN only)		
ACE-CL-208 (NCT02717611)	2	60	Adults with R/R CLL who are intolerant of ibrutinib	Acalabrutinib	Active, not recruiting	(53)
NCT02337829	2	48	Adults with R/R or TN CLL/SLL with del(17p)	Acalabrutinib	Active, not recruiting	N/A
ASCEND/ACE-CL-309 (NCT02970318)	3	306	Adults with R/R CLL	Acalabrutinib	Active, not recruiting	(22)
				BR		
				Idelalisib + R		
ACE-CL-001 (NCT02029443)	1/2	306	Adults with TN or R/R CLL/SLL/PLL or RT	Acalabrutinib	Active, not recruiting	(40, 48–50, 52, 63)
ELEVATE RR/ACE-CL-006 (NCT02477696)	3	533	Adults with R/R CLL with del(17p) or del(11q)	Acalabrutinib	Active, not recruiting	N/A
				Ibrutinib		
ELEVATE TN CLL/ACE-CL-007 (NCT02475681)	3	535	Adults with TN CLL	Acalabrutinib	Active, not recruiting	(21)
				Acalabrutinib + G		
				G + chlorambucil		
CLL2-BAGG (NCT03787264)	2	46	Adults with R/R CLL	Sequential B, G, acalabrutinib, Ven	Active, not recruiting	N/A
ACE-CL-311 (NCT03836261)	3	780	Adults with TN CLL without del(17p) or mutated *TP53*	Acalabrutinib + Ven	Recruiting	N/A
				Acalabrutinib + Ven ± G		
				FCR or BR		
ACE-CL-110 (NCT03328273)	1/2	62	Adults with R/R CLL	Acalabrutinib + AZD6738	Recruiting	N/A
AVO (NCT03580928)	2	37	Adults with TN CLL	Acalabrutinib + Ven + G	Recruiting	N/A
NCT03516617	2	120	Adults with TN CLL/SLL	Acalabrutinib ± G	Recruiting	N/A
NCT03788291	2	40	Adults with TN CLL/SLL	Acalabrutinib + R	Recruiting	N/A
ASSURE (NCT04008706)	3b	600	Adults with CLL, 4 cohorts: TN, R/R, prior BTKi therapy, and concomitant vitamin K antagonists	Acalabrutinib	Recruiting	N/A
NCT04178798	3	130	Adults with early stage CLL with high risk of early disease progression	Acalabrutinib vs watch and wait	Recruiting	N/A
NCT03868722	2/3	212	Adults with TN CLL at high risk of infection	Acalabrutinib + Ven	Recruiting	N/A
NCT04075292	3	150	Adults TN CLL	Acalabrutinib vs chlorambucil + R	Recruiting	N/A
NCT04169737	2	168	High risk, recurrent, or refractory CLL or SLL	Acalabrutinib + Ven ± early G	Not yet recruiting	N/A
NCT04505254	2	60	Adults with TN CLL	Acalabrutinib + G	Not yet recruiting	N/A
REVEAL (NCT04523428)	2	60	Adult patients with R/R CLL or SLL who had relapsed after first line Ven + anti-CD20	Acalabrutinib + Ven	Not yet recruiting	N/A
**Other hematological indications**						
ACE-MY-001 (NCT02211014)	1b	28	Adults with MM	Acalabrutinib	Completed	N/A
ACE-WM-001 (NCT02180724)	2	106	Adults with WM	Acalabrutinib	Active, not recruiting	(64)
ACE-LY-110 (NCT03205046)	1/2	25	Adults with R/R B-cell malignancies	Acalabrutinib + vistusertib	Active, not recruiting	N/A
ACE-LY-001 (NCT02328014)	1/2	40	Adults with B-cell malignancies	Acalabrutinib +	Active, not recruiting	(54)
				ACP-319		
ACE-LY-002 (NCT02112526)	1	21	Adults with *de novo* activated B-cell subtype of DLBCL	Acalabrutinib	Active, not recruiting	N/A
KEYNOTE145/ACE-LY-005 (NCT02362035)	1/2	159	Adults with hematological malignancies	Acalabrutinib + pembrolizumab	Active, not recruiting	(55)
ACCEPT (NCT03571308)	2	39	Patients (≥16 years) with DLBCL	Acalabrutinib + R-CHOP	Active, not recruiting	N/A
D8220C00001 (NCT03198650)	1	25	Japanese adults with advanced B-cell malignancies	Acalabrutinib	Recruiting	N/A
PRISM Study (NCT03527147)	1	42	Adults with R/R aggressive NHL	Acalabrutinib + AZD9150	Recruiting	N/A
				Acalabrutinib + AZD6738		
				Acalabrutinib + Hu5F9-G4 + R		
				Acalabrutinib + AZD5153		
ACE-LY-003 (NCT02180711)	1/2	126	Adults with B-cell NHL	Acalabrutinib	Recruiting	N/A
				Acalabrutinib + R		
				Acalabrutinib + lenalidomide + R		
NCT04002947	2	112	Adults with TN DLBCL	Acalabrutinib + DA-EPOCH	Recruiting	N/A
				Acalabrutinib + R-CHOP		
NCT03736616	2	47	Adults with R/R DLBCL	Acalabrutinib + RICE	Recruiting	N/A
NCT04094142	2	66	Adults with R/R B-cell NHL	Acalabrutinib with R + lenalidomide	Recruiting	N/A
NCT03932331	1/2	105	Chinese adults with R/R MCL, CLL, and other B-cell malignancies	Acalabrutinib	Recruiting	N/A
NCT04404088	24	2	Adults with CD20+ stage III–IV, grade 1–3a TN FL	Acalabrutinib + lenalidomide + R	Recruiting	N/A
NCT04462328	21	1	Adults with CNS lymphoma	Acalabrutinib + durvalumab	Not yet recruiting	N/A
NCT04502394	1b/2	84	Adults with R/R lymphoma (CLL; DLBCL; B-cell NHL)	Acalabrutinib + KRT-232	Not yet recruiting	N/A
NCT04257578	20	1/2	Adults with B-cell lymphoma	Acalabrutinib + axicabtagene ciloleucel	Not yet recruiting	N/A
STELLAR (NCT03899337)	2	105	Adults with newly diagnosed RT	R-CHOP	Not yet recruiting	N/A
				Acalabrutinib + R-CHOP		
NCT04189952	2	46	Adults with R/R lymphoma (DLBCL; CLL; SLL; MZL)	Acalabrutinib + RICE	Not yet recruiting	N/A
NCT04337827	2	62	Adults with newly diagnosed PTLD	Acalabrutinib + R	Not yet recruiting	N/A

BR, bendamustine and rituximab; BTKi, Bruton tyrosine kinase inhibitor; CLL, chronic lymphocytic leukemia; CNS, central nervous system; CR, cytarabine and rituximab; DA-EPOCH, infusional rituximab, cyclophosphamide, doxorubicin, etoposide, vincristine, and prednisone; DLBCL, diffuse large B-cell lymphoma; FCR, fludarabine, cyclophosphamide, and rituximab; FL, follicular lymphoma; G, obinutuzumab; MCL, mantle cell lymphoma; MM, multiple myeloma; MZL, marginal zone lymphoma; N/A, not available; NHL, non-Hodgkin lymphoma; PLL, prolymphocytic leukemia; PTLD, post-transplant lymphoproliferative disease; R, rituximab; R-CHOP, rituximab, cyclophosphamide, vincristine, doxorubicin, and prednisolone; RICE, rituximab, ifosfamide, carboplatin, and etoposide; R/R, relapsed/refractory; RT, Richter’s transformation; SLL, small lymphocytic leukemia; TN, treatment-naïve; Ven, venetoclax; WM, Waldenstrom’s macroglobulinemia.

A search on ClinicalTrials.gov for ‘acalabrutinib’ and ‘ACP-196’ was conducted on 27 August 2020. Trials in indications outside of hematological malignancies were not included because they are not within the scope of this review.

a Estimated enrollment is used when actual enrollment is not available.

B-cell lymphoma-2 (BCL-2) proteins regulate the intrinsic mitochondrial apoptotic pathway and represent another therapeutic target in B-cell malignancies (66). Venetoclax is an approved, highly selective BCL-2 inhibitor used in treatment of patients with CLL. In ACE-CL-311, an ongoing, randomized, open-label phase 3 study with a target enrollment of 780 patients with treatment-naïve CLL without del(17p) or mutated *TP53*, acalabrutinib in combination with the BCL-2 inhibitor venetoclax with and without obinutuzumab *versus* chemotherapy will be evaluated (expected completion in 2024) (67). A randomized, phase 3, double-blind trial is recruiting patients with MCL (ACE-LY-308; NCT02972840) to investigate bendamustine/rituximab alone or combined with acalabrutinib in treatment-naïve patients (expected completion in 2023).

Encouraging safety and efficacy results have been demonstrated in an ongoing phase 2 study of patients with CLL given combined acalabrutinib, venetoclax, and obinutuzumab (AVO) (NCT03580928). Of 36 patients with at least 16 months of follow-up at the interim analysis, the ORR was 100%; 78% of patients—in a population comprising nearly 40% of patients with TP53 mutations—had bone marrow-undetectable MRD by 15 months of time-limited therapy (68). In a phase 1b study of relapsed/refractory or treatment-naïve CLL patients (NCT02296918) (*n* = 12 per cohort), 92% and 83% of patients, respectively, remained on a modified AVO combination treatment (acalabrutinib plus obinutuzumab/rituximab plus venetoclax) at the median follow-up time of 23.2 and 22.0 months. AEs of the combined treatment were similar to those of the individual agents. After 16 treatment cycles, the ORR for relapsed/refractory patients was 92% and for treatment-naïve patients was 100% (69).

## Discussion

Results of clinical trials in patients with relapsed/refractory MCL, relapsed/refractory and treatment-naïve CLL, or WM demonstrate that acalabrutinib is well tolerated and effective. Acalabrutinib is also well tolerated in high-risk patients with CLL, including those with del(17p) and mutated *TP53* (21, 45) and those who are intolerant to ibrutinib (52). The improved selectivity profile of acalabrutinib, compared with ibrutinib, provides the potential for a reduced risk of toxicity, which may result in improved treatment outcomes and decreased rates of discontinuation.

The treatment landscape for patients with CLL and other B-cell malignancies is rapidly evolving away from chemoimmunotherapy-based treatment to targeted treatment and combinations. For example, BTK has been shown to modulate PD-1 expression in DLBCL cells *in vitro*, which raises the possibility that combinations of BTK inhibitors with immune checkpoint inhibitors may increase efficacy (70). A clinical trial assessing avelumab (anti-PDL1) combined with ibrutinib for patients with relapsed/refractory DLBCL or MCL is currently underway (NCT03440567).

New targeted therapies are available for the treatment of B-cell malignancies, including BTK inhibitors, the BCL-2 inhibitor venetoclax, anti-CD20 mAbs (obinutuzumab, ofatumumab), immunomodulatory agents (*e.g.* lenalidomide), and PI3K inhibitors (*e.g.* idelalisib). Additionally, trials of combined targeted therapy with agents aiming toward fixed-duration treatment to achieve deep and durable unmaintained remission in patients with CLL are underway (71). Recent results showed targeted therapies are more effective than traditional chemoimmunotherapy, changing the standard of care in B-cell malignancies. For example, the ALLIANCE phase 3 trial showed superior PFS with ibrutinib and ibrutinib plus rituximab compared with bendamustine plus rituximab in older treatment-naïve patients with CLL; the addition of rituximab to ibrutinib did not provide additional PFS benefit compared to ibrutinib alone (10). Similarly, the E1912 phase 3 trial showed that ibrutinib plus rituximab was superior to fludarabine, cyclophosphamide, and rituximab, with respect to PFS and OS in treatment-naïve patients with CLL (11). Results of iLLUMINATE showed that ibrutinib plus obinutuzumab resulted in superior PFS and OS compared with chlorambucil plus obinutuzumab in treatment-naïve patients with CLL (72). Additionally, findings of ELEVATE-TN indicated superior PFS rates of both acalabrutinib-containing arms compared with obinutuzumab–chlorambucil (21). With venetoclax, PFS and OS were superior to bendamustine plus rituximab in patients with relapsed/refractory CLL in the phase 3 MURANO trial (73). Results of the phase 3 CLL14 trial indicated that patients with treatment-naïve CLL receiving venetoclax plus obinutuzumab had a significantly longer PFS than did patients on chlorambucil plus obinutuzumab (HR, 0.31; 95% CI: 0.22–0.44; *p* < 0.0001) (74). Interim results from an ongoing phase 2 trial evaluating a time-limited combination of acalabrutinib, venetoclax, and obinutuzumab in treatment-naïve CLL (NCT03580928) showed a 100% response rate (75% PR; 25% CR) (75).

Results of a recent network meta-analysis comparing the outcomes of acalabrutinib plus obinutuzumab (ELEVATE-TN), ibrutinib plus obinutuzumab (iLLUMINATE), and venetoclax plus obinutuzumab (CLL14) in treatment-naïve patients with CLL suggested that PFS improvement was greatest in patients treated with acalabrutinib plus obinutuzumab (76). A head-to-head comparison of acalabrutinib *versus* ibrutinib is ongoing. It will be interesting to see results of ongoing phase 3 trials with acalabrutinib and whether the improved tolerability translates to a real-world clinical benefit.

## Conclusion

BTK inhibitors have remarkably advanced treatment options for patients with B-cell malignancies; hence, BTK is a proven critical therapeutic target. Factors driving further development of BTK inhibitors have been the toxicities and tolerability issues noted with the first-in-class compound, ibrutinib, and the patterns and factors associated with resistance, most notably mutations in C481 of BTK, the binding site of the covalent inhibitors. Off-target kinase inhibition is thought to contribute to the toxicity profile of individual agents, so focusing on compounds with narrower kinome profiles of inhibition may reduce toxicity. Tolerability and toxicities can heavily impact the ability to deliver an intended effective dose and maintain patients on treatment. The covalent BTK inhibitors—particularly ibrutinib and acalabrutinib—have some toxicities in common, and each has circumstances and conditions that might limit use. The experience of the authors has been that fewer patients require dose adjustment or treatment discontinuation with acalabrutinib compared with ibrutinib. Head-to-head randomized trials, such as the recently completed ELEVATE RR/ACE-CL-006 trial (NCT02477696), are needed to confirm differences in tolerability. The efficacies of the covalent BTK inhibitors appear to be similar; however, long-term differences may be revealed related to tolerability. Acalabrutinib was tolerated in patients who had discontinued ibrutinib because of side effects, importantly allowing for longer on-target treatment (77). Further, acalabrutinib had reported activity in patients with CLL who progressed on ibrutinib (52).

Given that treatment with BTK inhibitors continues until disease progression, and as these agents are highly effective—providing many years of disease control—consideration must be given for longer exposure time and follow-up with ibrutinib-based studies compared with those of other BTK inhibitors. Reversible third-generation inhibitors of BTK are in development, with early reports indicating activity and excellent tolerability in patients who are refractory to a covalent BTK inhibitor.

Finally, fixed durations of treatment, with long remissions off-treatment, have been made possible by the very deep remissions observed with venetoclax-based treatment. The authors believe that fixed-duration targeted therapy, including BTK and BCL-2 inhibitors, will be the next standard of care.

## Author Contributions

WW and HA contributed to the writing and editing of this manuscript. Both authors also reviewed and approved the final draft for submission and take responsibility for all the content.

## Funding

Medical writing support was provided by Adele Buss, PhD, of Oxford PharmaGenesis, Oxford, UK, and was funded by AstraZeneca LP.

## Conflict of Interest

WW has received research funding from AbbVie, Acerta Pharma, Emergent, Genentech, Gilead, GlaxoSmithKline/Novartis, Janssen, Juno, Karyopharm, Kite, and Pharmacyclics.

The remaining author declares that the research was conducted in the absence of any commercial or financial relationships that could be construed as a potential conflict of interest.

The authors declare that this study received funding from Astra Zeneca LP. The funder had the following involvement in the study: medical writing support.

## Abbreviations

AE, adverse event; BTK, Bruton tyrosine kinase; cGVHD; chronic graft *versus* host disease; CI, confidence interval; CLL, chronic lymphocytic leukemia; CR, complete response; DLBCL, diffuse large B-cell lymphoma; DOR, duration of response; ECOG, Eastern Cooperative Oncology Group; EGF, epidermal growth factor; FDA, Food and Drug Administration; GCB, germinal center B-cell; IGHV, immunoglobulin heavy-chain variable; MCL, mantle cell lymphoma; NHL, non-Hodgkin lymphoma; ORR, overall response rate; OS, overall survival; PD-1, programmed cell death protein 1; PFS, progression-free survival; PI3Kδ, phosphatidyl-3-kinase delta; PK, pharmacokinetic; PR, partial response; SLL, small lymphocytic leukemia; WM, Waldenstrom’s macroglobulinemia.
